# Intestinal Barrier Dysfunction and Stem Cell Impairment Following Cardiac Surgery in Pigs: A Porcine Model Study

**DOI:** 10.3390/biology15120930

**Published:** 2026-06-15

**Authors:** Haoyang Nian, Yaqi Li, Zhihao Chen, Jianping Zhu, Ping Yang, Li Cui

**Affiliations:** 1 Department of Animal Science, School of Agriculture and Biology, Shanghai Jiao Tong University, Shanghai 200240, China; 2 Shanghai Key Laboratory of Veterinary Biotechnology, Shanghai Jiao Tong University, Shanghai 200240, China; 3 Covidien (Shanghai) Management Consulting Company, Gumai Road, Xuhui District, Shanghai 200240, China; 4 PharmaLegacy Laboratories (Shanghai) Co., Ltd., Longgui Road, Pudong New Area, Shanghai 200240, China

**Keywords:** organoid, intestinal stem cells, cardiac surgery, NF-κB

## Abstract

Cardiac surgery induces systemic stress responses that may compromise intestinal homeostasis in animal patients, yet the underlying mechanisms remain poorly understood. This study investigated postoperative intestinal pathophysiology in a porcine model, focusing on the activation of the NF-κB pathway and its impact on intestinal stem cell function. Thirty-two large white pigs (40–50 kg) were randomized into control and model groups; the model group underwent simulated cardiac procedures involving aortotomy and left ventricular outflow tract exposure under standardized anesthesia. Our findings revealed significant NF-κB pathway activation and upregulation of matrix metalloproteinases (MMPs) in the intestine following surgery. Intestinal stem cells isolated from crypts showed downregulated expression of key barrier proteins. Notably, organoids derived from the model group exhibited impaired early proliferative capacity and delayed morphogenesis, despite preserved differentiation potential upon extended culture. This suggests a stress-induced state that favors the maintenance of stemness over commitment to differentiation. These results delineate an NF-κB/MMP-mediated mechanism linking surgical stress to intestinal barrier dysfunction and stem cell dysregulation in this large animal model. Crucially, the study provides insights that may help improve perioperative care and reduce gastrointestinal complications in animal patients undergoing major surgeries, thereby enhancing animal welfare. The identified pathways offer potential targets for developing supportive therapies in veterinary practice.

## Simple Summary

This study investigated the intestinal response to surgical stress induced by cardiac procedures in a porcine model. Thirty-two large white pigs were randomized into control and model groups, with the latter undergoing simulated cardiac surgery. The results demonstrated significant postoperative activation of the NF-κB pathway and upregulation of matrix metalloproteinases in intestinal tissue. The isolation of intestinal stem cells from crypts revealed a downregulation of key barrier proteins. Intestinal organoids derived from the model group exhibited reduced proliferative capacity and delayed morphogenesis during early culture, reflecting transient injury effects on stem cells. However, these impairments did not affect the differentiation potential of crypt-derived stem cells, which remained intact after extended culture. Using organoids as an in vitro complement to in vivo analysis provides a platform for further mechanistic studies. It may also reduce the need for additional animal-based validation and support ethical research practices that prioritize animal welfare. These findings indicate that cardiac surgery in swine triggers an NF-κB/MMP-mediated mechanism contributing to intestinal barrier dysfunction and stem cell dysregulation in this animal model.

## 1. Introduction

Cardiac surgery is a major clinical intervention associated with substantial systemic stress, inflammatory activation, and oxidative injury, particularly in complex procedures involving cardiopulmonary bypass and ischemia–reperfusion [1,2,3]. These perioperative disturbances may contribute not only to myocardial injury but also to postoperative dysfunction in distant organs. Gastrointestinal complications after cardiac surgery are relatively uncommon but clinically important because they are associated with considerable morbidity and poor outcomes [4,5]. Therefore, clarifying the mechanisms of intestinal injury after cardiac surgery is of clear clinical relevance.

The intestine is one of the most metabolically active organs and plays a central role in nutrient absorption and barrier defense [6]. Because of its high metabolic demand and dependence on adequate perfusion, it may be particularly vulnerable to perioperative stress during cardiac surgery. Intestinal hypoperfusion may lead to epithelial barrier dysfunction [7], followed by dysbiosis and bacterial translocation [8,9]. In addition, systemic inflammation after cardiac surgery has been associated with a reduction in beneficial bacteria and an increase in pro-inflammatory bacteria, which may further aggravate intestinal inflammation [10,11]. Matrix metalloproteinases (MMPs), a family of zinc-dependent endopeptidases involved in the regulation of cytokines, chemokines, and growth factors [12], have also been implicated in tissue injury during cardiac surgery. In particular, MMP-2 and MMP-9 have been associated with cardiac-surgery-related tissue damage [13,14], and circulating MMPs may contribute to injury in distant organs, including the intestine [15]. Given the susceptibility of the intestinal mucosa to ischemia–reperfusion injury [16], these perioperative insults may ultimately compromise intestinal barrier integrity and epithelial homeostasis [17].

In addition to barrier function, intestinal homeostasis depends on the continuous proliferation and differentiation of intestinal stem cells. These stem cells give rise to multiple epithelial lineages, including goblet cells, enteroendocrine cells, Paneth cells, and absorptive cells [18,19], thereby maintaining epithelial renewal and repair. With the development of three-dimensional self-organizing organoid culture systems, intestinal organoids have become physiologically relevant in vitro models for studying epithelial biology [20]. Because organoids recapitulate key features of intestinal epithelial organization and differentiation, they provide a useful platform for evaluating whether cardiac surgery-associated stress affects not only barrier-related alterations but also stem cell integrity and regenerative capacity.

Although intestinal injury after cardiac surgery has received increasing attention, the mechanisms linking systemic stress, intestinal barrier dysfunction, and stem cell impairment remain insufficiently understood. In particular, it is still unclear whether the intense perioperative stress associated with cardiac surgery alters the proliferative and differentiation capacities of intestinal stem cells in a clinically relevant large animal model. Therefore, the present study was designed to investigate cardiac-surgery-related intestinal injury in pigs, with a particular focus on barrier dysfunction, stemness maintenance, and epithelial differentiation, and to further examine these changes using intestinal organoid cultures.

## 2. Materials and Methods

### 2.1. Ethics Statement

All the animal experiments were reviewed and approved by the Institutional Animal Care and Use Committee of Shanghai (Shanghai, China; approval no. MICSH-IACUC-2024-008; approval date: 7 May 2024). All the procedures were carried out in accordance with the National Research Council Guide for the Care and Use of Laboratory Animals.

### 2.2. Animals and Management

A total of 32 large white pigs (40–50 kg) were randomly divided into two groups, including the control group and the model group. The pigs in the model group underwent surgical simulations designed to replicate cardiac procedures through the following operative sequence: The protocol commenced with an aortotomy at the left-noncoronary commissure. A precision-engineered Y-shaped incision was extended through the aortic valve, carefully dissecting both the left coronary and noncoronary cusps to their respective nadirs. This approach allowed full exposure of the left ventricular outflow tract (LVOT). Throughout the procedure, gentle tissue manipulations were performed under continuous hemodynamic monitoring, with particular attention to maintaining physiological transvalvular pressure gradients. Anesthetic management followed a standardized protocol: Premedication consisted of Zoletil^®^ (8 mg/kg, intramuscular administration) followed by induction with isoflurane (4–5% concentration) via face mask. Orotracheal intubation was achieved using 7–7.5 mm endotracheal tubes, with subsequent maintenance of general anesthesia through vaporized isoflurane (1.5–3% concentration) delivered via mechanical ventilation. The pigs in the control group underwent a sham operation under the same anesthesia, surgical manipulation, operative environment, instrument conditions, and perioperative management as the model group, but without the ischemia–reperfusion procedure. Except for the ischemia–reperfusion modeling step, all the procedures, including intraoperative vital sign maintenance, postoperative care, and sample collection, were identical between the two groups. At the end of the experiment, the animals in both groups were deeply anesthetized with an intravenous injection of Zoletil (7–10 mg/kg) and euthanized by bloodletting. In this study, four pigs were randomly selected from each group of 16 using a computer-generated random number method to serve as representative samples.

### 2.3. Serum Sampling and Detection

Blood samples (5 mL) were collected and left to stand for 4 h. The supernatant was then centrifuged at 3000 rpm for 10 min at room temperature. The serum was transferred to 1.5 mL microcentrifuge tubes and stored at −20 °C. Corticotrophin (ACTH; minimum detection limit, 1 pg/mL) was measured using a commercial ELISA kit (Fenxi Biotechnology Co., Ltd., Shanghai, China) following the manufacturer’s instructions. The intra-assay coefficient of variation was less than 15%, and the inter-assay coefficient of variation was below 15%. Sample absorbance (OD) was read with an enzyme-labeling instrument, and the standard curve was generated based on the concentrations and OD values of the standard samples.

Myocardial injury markers were also assessed during the experiment. Creatine kinase (CK; minimum detection limit, 6 U/L) was measured using a commercial kit (Roche Diagnostics GmbH, Mannheim, Germany). The intra-assay coefficient of variation was <9.6%, and the inter-assay coefficient of variation was <8%. Creatine kinase-MB (CK-MB; minimum detection limit, 24 U/L) was measured using a commercial kit (Maccura Biotechnology Co., Ltd., Chengdu, China). The intra-assay coefficient of variation was <5%, and the inter-assay coefficient of variation was <10%. Myoglobin (MYO; minimum detection limit, 1.0 ng/mL) was measured using a commercial kit (Mindray Bio-Medical Electronics Co., Ltd., Shenzhen, China). The intra-assay coefficient of variation was <6%, and the inter-assay coefficient of variation was <10%. Elecsys Troponin T hs STAT (cTnT; minimum detection limit, 3.0 ng/L) was measured using a commercial kit (Roche Diagnostics GmbH, Mannheim, Germany). The intra-assay coefficient of variation was <8%, and the inter-assay coefficient of variation was <10%. Lactate dehydrogenase (LDH; minimum detection limit, 5 U/L) was measured using a commercial kit (Mindray Bio-Medical Electronics Co., Ltd., Shenzhen, China). The intra-assay coefficient of variation was <4%, and the inter-assay coefficient of variation was <6.8%.

### 2.4. Tissue Sampling

After euthanasia, the duodenum was collected, flash-frozen in liquid nitrogen, and then stored at −80 °C for subsequent analysis.

### 2.5. Intestinal Crypt Isolation

Porcine duodenal crypts were isolated from animals in both the control and model groups. The intestinal tissues were collected immediately after euthanasia and then divided into 2–4 cm pieces. The villi and mucosal layer were removed with a microscope slide before the tissue was further cut into 5 mm fragments. The tissue fragments were rinsed five times with ice-cold PBS and then incubated in 2.5 mM EDTA for 45 min at 4 °C. The crypts were then released by vigorous pipetting in 10 mL of ice-cold PBS.

### 2.6. Organoid Culture and Passage

The crypt-containing suspensions were first pelleted by centrifugation at 400× *g* for 5 min. The crypts were washed twice with ice-cold PBS and then resuspended in Matrigel (Corning, New York, NY, USA). Aliquots of 50 μL of the crypt–Matrigel mixture were seeded into each well of a 24-well plate pre-warmed to 37 °C. After gelation at 37 °C for 15 min to allow the Matrigel to solidify, 800 μL of PIO medium was added to each well. The organoids were cultured at 37 °C in an atmosphere of 5% CO_2_. The composition of the PIO medium is shown in Supplementary Table S1. The culture medium was refreshed every 4 days.

For organoid passage, the Matrigel domes were broken up with PIO medium, and the resulting suspension was transferred to a 15 mL centrifuge tube and spun at 400× *g* for 5 min. Most of the Matrigel and supernatant were removed, and the organoids were incubated with 1 mL of TrypLE Express (Invitrogen, Carlsbad, CA, USA) at 37 °C for 5 min. Digestion was stopped by adding 5 mL of basal medium (Advanced DMEM/F12, Invitrogen, USA). After centrifugation at 400× *g* for 5 min, the supernatant was discarded. The organoids were then resuspended in Matrigel, and 50 μL of the organoid–Matrigel mixture was plated into each well of another pre-warmed 24-well plate. After the Matrigel had solidified, 800 μL of PIO medium was added to each well, and the organoids were further cultured at 37 °C in 5% CO_2_.

### 2.7. Growth of Organoids and High-Throughput Analysis

The growth monitoring of the organoids was performed using a high-throughput standardized imaging analysis system (CountStar Castor S1, Shanghai, China). Briefly, the 24-well plate was placed into the chamber, the scanning interval was set to 0.02 mm per layer, and the total scanning distance was set to 1.2 mm. Bright-field images were collected each day. The quantitative analysis of the morphology and phenotype of the organoids was performed using Castor S1.

### 2.8. Quantitative Real-Time PCR Analysis

Total RNA from the duodenum and organoids was extracted using the RNAeasy Animal RNA Isolation Kit (Beyotime). RNA quantity and quality were determined using a micro-UV spectrophotometer (Eppendorf, Hamburg, Germany), and cDNA synthesis was performed with Revertra Ace (Toyobo, Tokyo, Japan). RT-PCR reactions were performed using a LightCycler^®^ 96 Instrument (Roche, Basel, Switzerland) with NovoStart SYBR qPCR SuperMix (Novoprotein, Suzhou, China). Expression levels were determined using the 2^−∆∆CT^ method and normalized to β-actin. The primers used in this study are listed in Supplementary Table S1.

### 2.9. Western Blot Analysis

Proteins were extracted from the duodenal tissues and organoids using lysis buffer for Western blotting and immunoprecipitation (Beyotime, Beijing, China) supplemented with protease inhibitor (Beyotime, Beijing, China). Protein concentration was measured with an enhanced BCA protein assay kit (Beyotime, Beijing, China). Equal amounts of protein were resolved by 10% or 12% SDS-PAGE and then transferred onto nitrocellulose membranes. After blocking with 5% BSA, the membranes were incubated with primary antibodies, followed by HRP-conjugated secondary antibodies. The primary antibodies used in this study and their dilution ratios are listed in Supplementary Table S2. Protein bands were visualized using a UVP Auto Chemi Image System (Tanon 4600SF, Tanon, Shanghai, China) after treatment with BeyoECL Star reagent (Beyotime, Beijing, China). Band intensities were quantified using ImageJ software (Version 1.8.0, NIH, Bethesda, MD, USA).

### 2.10. Immunofluorescence

Organoid immunofluorescence staining was carried out as previously described [21]. Immunofluorescence images of the organoids were analyzed using ImageJ software (NIH, Bethesda, MD, USA). In each captured field, one intact organoid with clear boundaries was selected as the region of interest (ROI) for fluorescence quantification. Organoids showing obvious collapse, rupture, overlap, or staining artifacts were excluded from the analysis. The fluorescence signal was quantified as mean fluorescence intensity (MFI). For each group, data from three independent experiments were used for statistical analysis.

### 2.11. Statistical Analysis

Normality was first assessed using the Shapiro–Wilk test. For comparisons between two groups, Student’s *t*-test was used. The results are presented as mean ± standard error of the mean (SEM). A value of *p* < 0.05 was considered statistically significant. All the analyses were conducted using SPSS software (Version 20.0, SPSS Inc., Chicago, IL, USA).

## 3. Results

### 3.1. Cardiac Surgery Caused Intense Stress to Pigs

**Figure 1:**
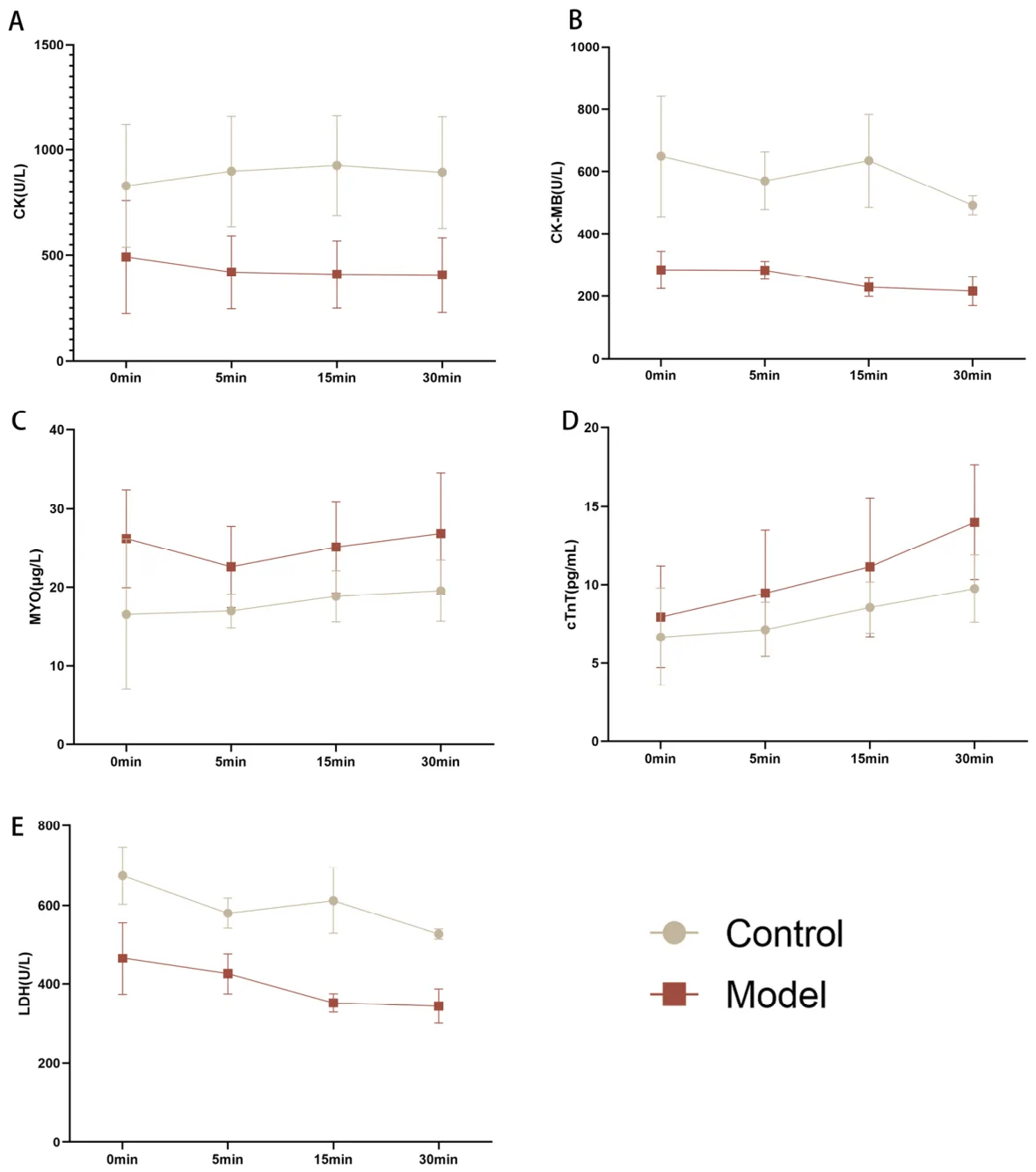
The dynamic changes in serum myocardial injury biomarkers during cardiac surgery. The serum levels of CK (**A**), CK-MB (**B**), MYO (**C**), cTnT (**D**), and LDH (**E**) were measured at 0, 5, 15, and 30 min after the initiation of the surgical procedure (or the corresponding sham procedure in the control group). The data are presented as mean ± SEM (*n* = 3 pigs per group).

As shown in Figure 1A–E, serum CK, CK-MB, MYO, cTnT, and LDH were measured at 0, 5, 15, and 30 min after the initiation of the surgical procedure (or the corresponding sham procedure in the control group). These myocardial injury biomarkers displayed dynamic intraoperative changes in both groups. Among them, MYO and cTnT showed progressive upward trends during the monitored period, and these increases tended to be more pronounced in the model group. In contrast, CK, CK-MB, and LDH showed more variable temporal profiles and did not exhibit the same increasing pattern. Statistical comparisons between the control and model groups were performed at each time point, but no significant differences were observed.

### 3.2. Cardiac Surgery Mediated Duodenum Intestine Barrier Disruption

**Figure 2:**
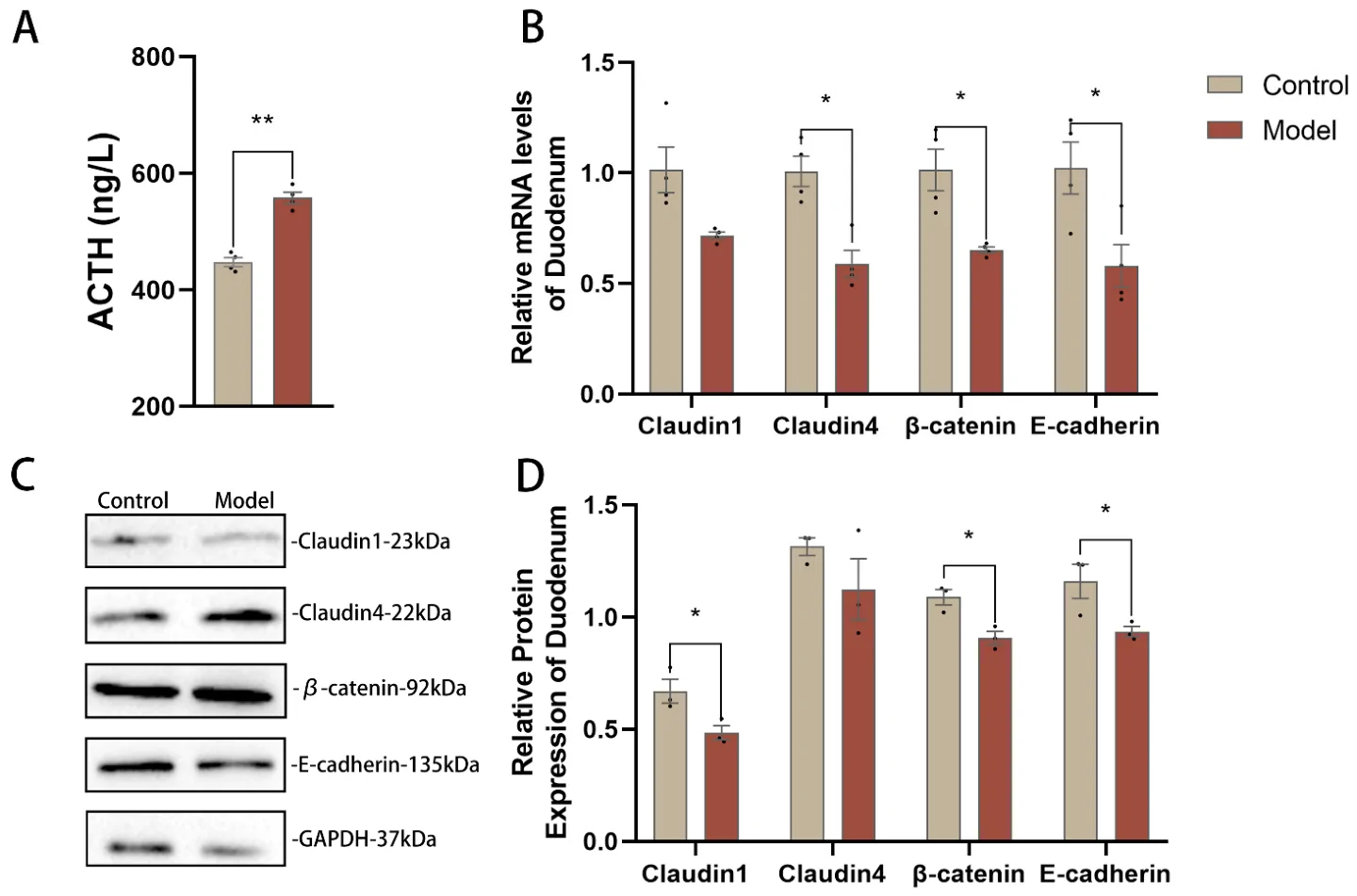
The effect of cardiac surgery on intestine barrier disruption. (**A**) shows the concentration of ACTH (ng/L), *n* = 4; (**B**) shows the relative mRNA levels of intestine-barrier-related genes, *n* = 4; (**C**,**D**) Western blotting was used to analyze the relative expression of Claudin1, Claudin4, β-catenin, and E-cadherin. Quantification was performed by measuring the intensity of each band by densitometry using ImageJ software, *n* = 3. The results are reported as mean ± SEM. * *p* < 0.05, ** *p* < 0.01. In this study, β-actin was selected as the reference in mRNA expression, and GAPDH was selected as the reference in protein expression. For each experiment, *n* refers to the number of independent biological replicates.

After the surgical procedures, the relative mRNA expression levels of intestinal barrier-related genes in the duodenum are shown in Figure 2B. The model group exhibited significant downregulation in the mRNA levels of these genes compared to the control group, with statistically significant reductions in Claudin4 (*p* = 0.0352), β-catenin (*p* = 0.0374) and E-cadherin (*p* = 0.0477). The relative protein expression levels in the duodenum showed the same trend; the expressions of Claudin1 (*p* = 0.0399), Claudin4 (*p* > 0.05), β-catenin (*p* = 0.0157) and E-cadherin (*p* = 0.0482) in the model group were lower than those in the control group. These findings collectively suggest that cardiac surgical procedures are associated with the transcriptional and translational attenuation of intestinal barrier integrity markers.

### 3.3. The NF-κB Pathway Was Activated During the Cardiac Surgery

**Figure 3:**
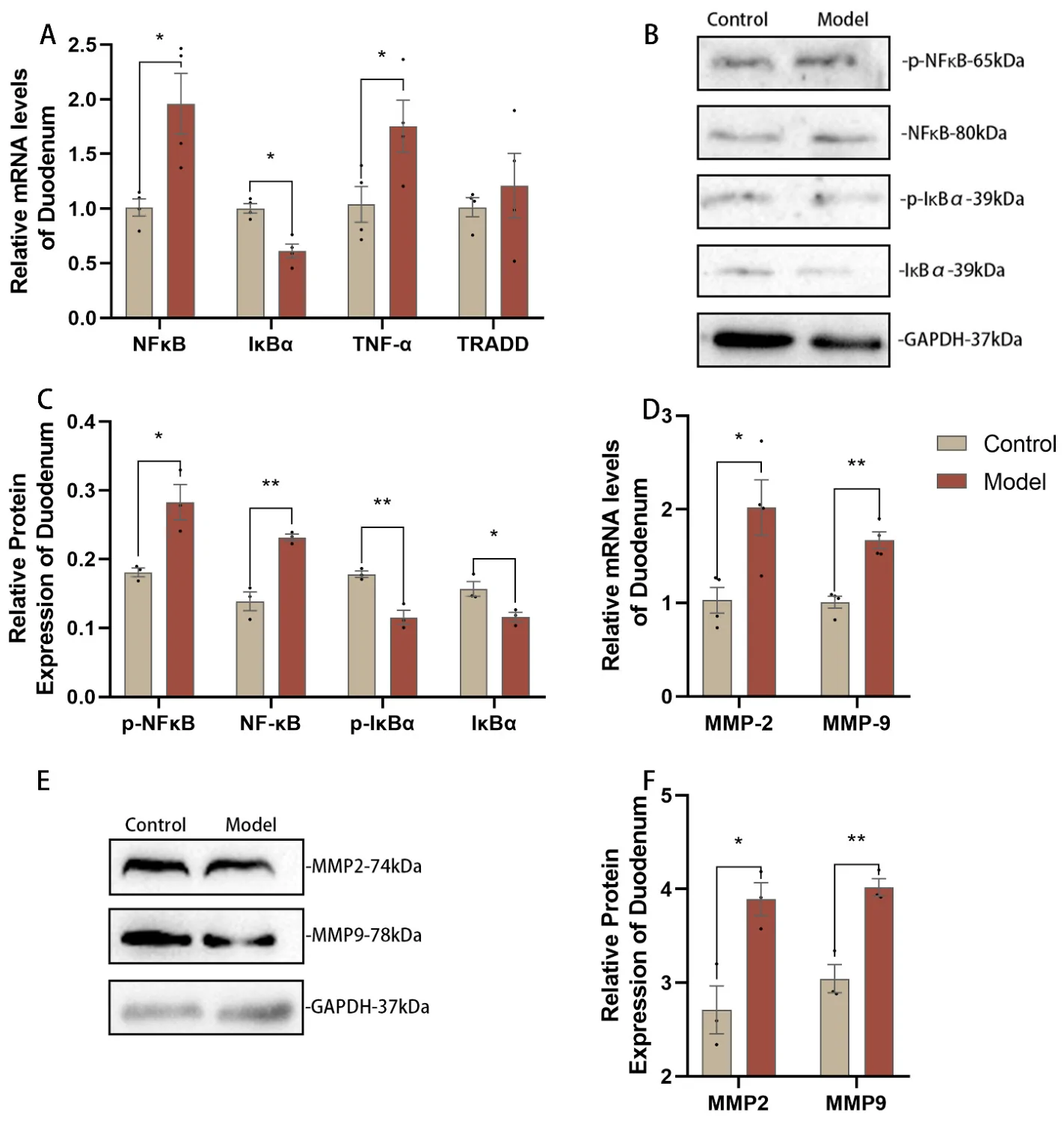
The NF-κB pathway was activated during the cardiac surgery. (**A**–**C**) shows the expression of NF-κB-pathway-related genes (*n* = 4) and proteins (*n* = 3); (**D**–**F**) shows both the mRNA (*n* = 4) and protein (*n* = 3) expressions of the MMPs. For each experiment, *n* refers to the number of independent biological replicates. The results are reported as mean ± SEM. * *p* < 0.05, ** *p* < 0.01.

To assess the effect of cardiac surgery on the NF-κB pathway, qPCR analysis was carried out first. The relative mRNA expression levels of NF-κB (*p* = 0.0357), TNF-α (*p* = 0.0490) and TRADD (*p* > 0.05) in the model group were higher than those in the control group (Figure 3A). Western blot analysis was then used to further evaluate NF-κB pathway activation. The results demonstrated that the relative expression levels of NF-κB (*p* = 0.0030) and p-NF-κB (*p* = 0.0183) were elevated in the model group compared to the control group, while both IκBα (*p* = 0.0339) and p-IκBα (*p* = 0.0051) showed reduced expression in the model group (Figure 3B,C). In line with these results, the model group exhibited increased mRNA expression levels of MMP-2 (*p* = 0.0272) and MMP-9 (*p* = 0.0038) relative to the control group (Figure 3D), which was corroborated by corresponding elevations in MMP-2 (*p* = 0.0190) and MMP-9 (*p* = 0.0052) protein expression (Figure 3E,F).

### 3.4. Cardiac-Surgery-Induced Intestinal Stress Modulates Intestinal Stem Cell Dynamics

**Figure 4:**
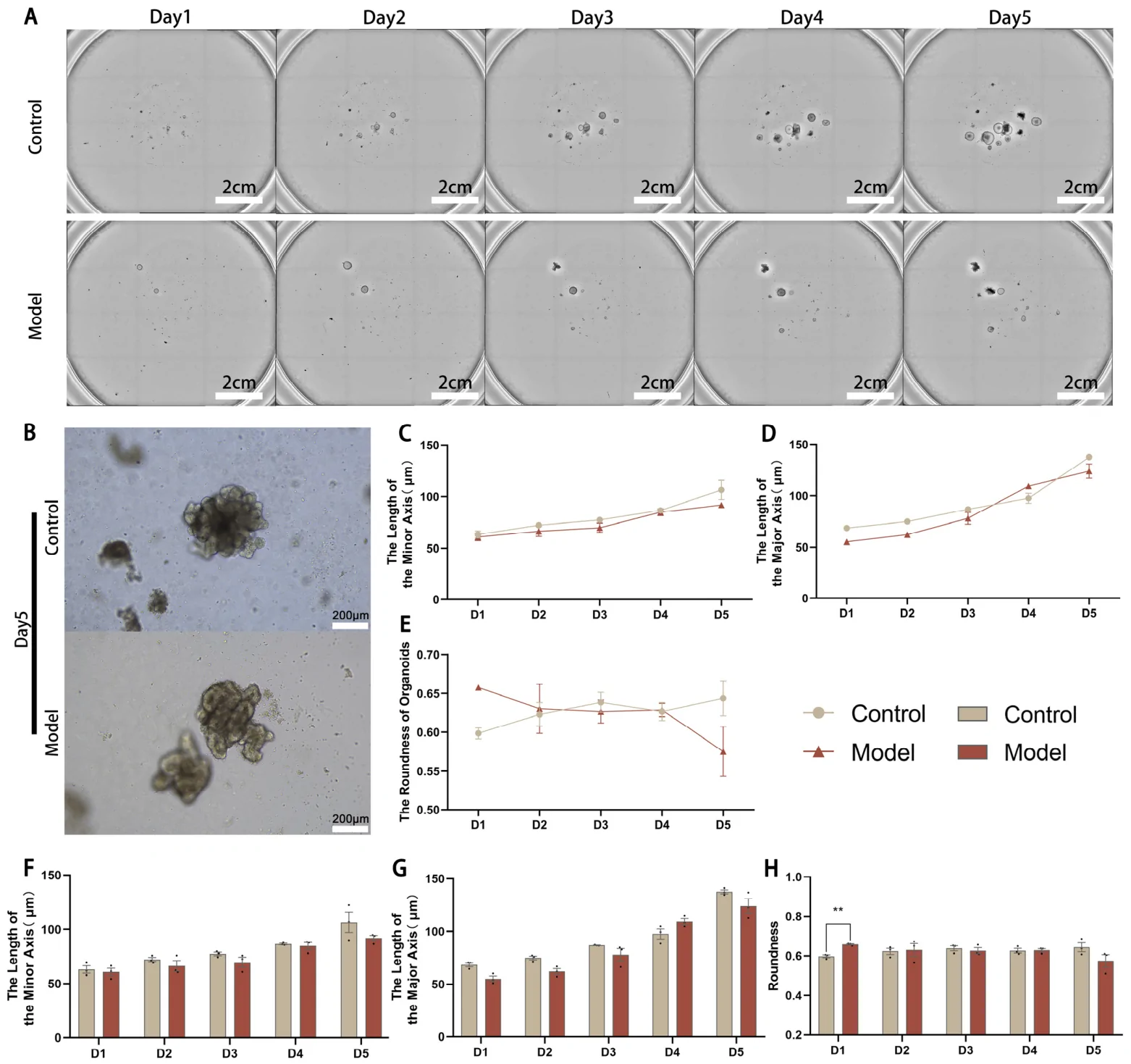
Cardiac-surgery-induced stress modulates intestinal stem cell dynamics. (**A**) shows the development of the organoids; (**B**), the organoids under a light microscope, the scale bar: 200 μm; (**C**–**E**), trends in the major axis, minor axis and roundness; (**F**–**H**), the quantitative analysis of the organoids’ growth. For each experiment, *n* refers to the number of independent biological replicates. The results are reported as mean ± SEM, *n* = 3. ** *p* < 0.01.

The intestinal organoids were cultured using the established protocol. Organoid growth was monitored and documented in both the control and model groups (Figure 4A,B). The model group exhibited delayed growth kinetics compared to the control group, and morphological observation showed greater differentiation in the control organoids after 5 days of Matrigel culture. The quantitative morphometric analysis (Figure 4F–H) showed trends toward reduced mean major and minor axis lengths in the model group relative to the controls, though these differences did not reach statistical significance. There was little overall difference in the roundness. However, organoid roundness was higher in the model group than in the control group on day 1 (*p* = 0.0038), indicating a more circular morphology at this time point.

### 3.5. Cardiac-Surgery-Induced Dysregulation of Intestinal Barrier Genes

**Figure 5:**
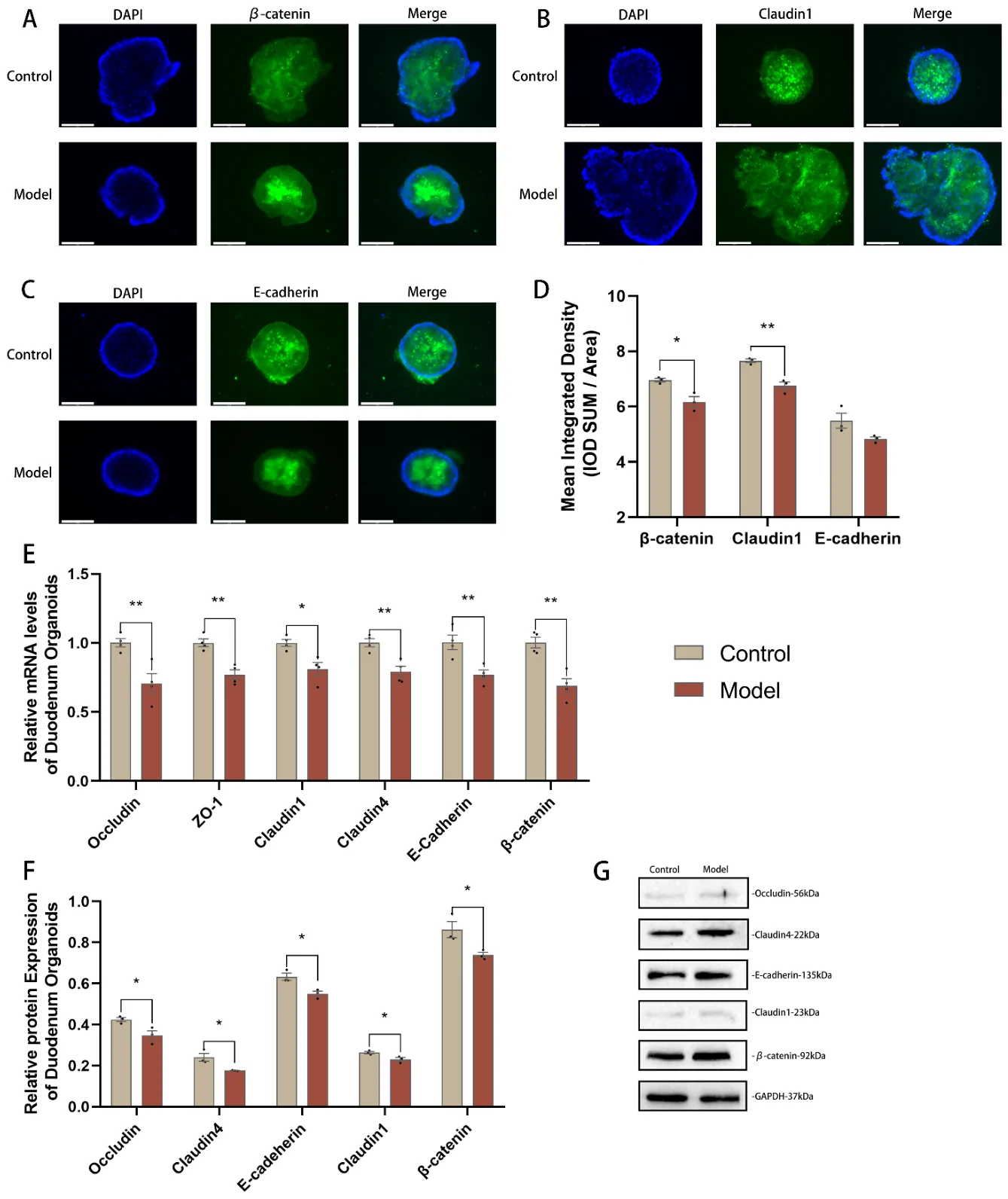
The expression of intestine barrier protein in intestine stem cells. (**A**–**C**), representative immunofluorescence images of the organoids labeled with DAPI (blue) and target protein antibodies (green), scale bar: 150 μm; (**D**), the quantification of protein relative expression using mean integrated density, *n* = 3; (**E**), the expression of intestine-barrier-related genes in the mRNA level, *n* = 4; (**F**,**G**), the relative protein expressions of duodenum organoids, *n* = 3. For each experiment, *n* refers to the number of independent biological replicates. The results are reported as mean ± SEM. * *p* < 0.05, ** *p* < 0.01.

Representative immunofluorescence images of intestinal-barrier-associated proteins are presented in Figure 5A–C. Quantitative analysis using mean integrated density showed significant reductions in β-catenin (*p* = 0.0174) and Claudin-1 (*p* = 0.0046) expression in the model group compared to the controls (Figure 5D). mRNA quantification (Figure 5E) revealed a marked downregulation of tight junction components in the model group, including Occludin (*p* = 0.0089), ZO-1 (*p* = 0.0022), Claudin1 (*p* = 0.0116), Claudin4 (*p* = 0.0055), E-cadherin (*p* = 0.0098) and β-catenin (*p* = 0.0029). Western blot analysis (Figure 5F,G) showed a similar pattern at the protein level, showing a reduced expression of Occludin (*p* = 0.0380), Claudin4 (*p* = 0.0288), E-cadherin (*p* = 0.0180), Claudin1 (*p* = 0.0351) and β-catenin (*p* = 0.0389) in the model group, thereby confirming impaired barrier integrity.

### 3.6. Cardiac-Surgery-Induced Stress Attenuates Intestinal Stem Cell Differentiation While Preserving Lineage Plasticity

**Figure 6:**
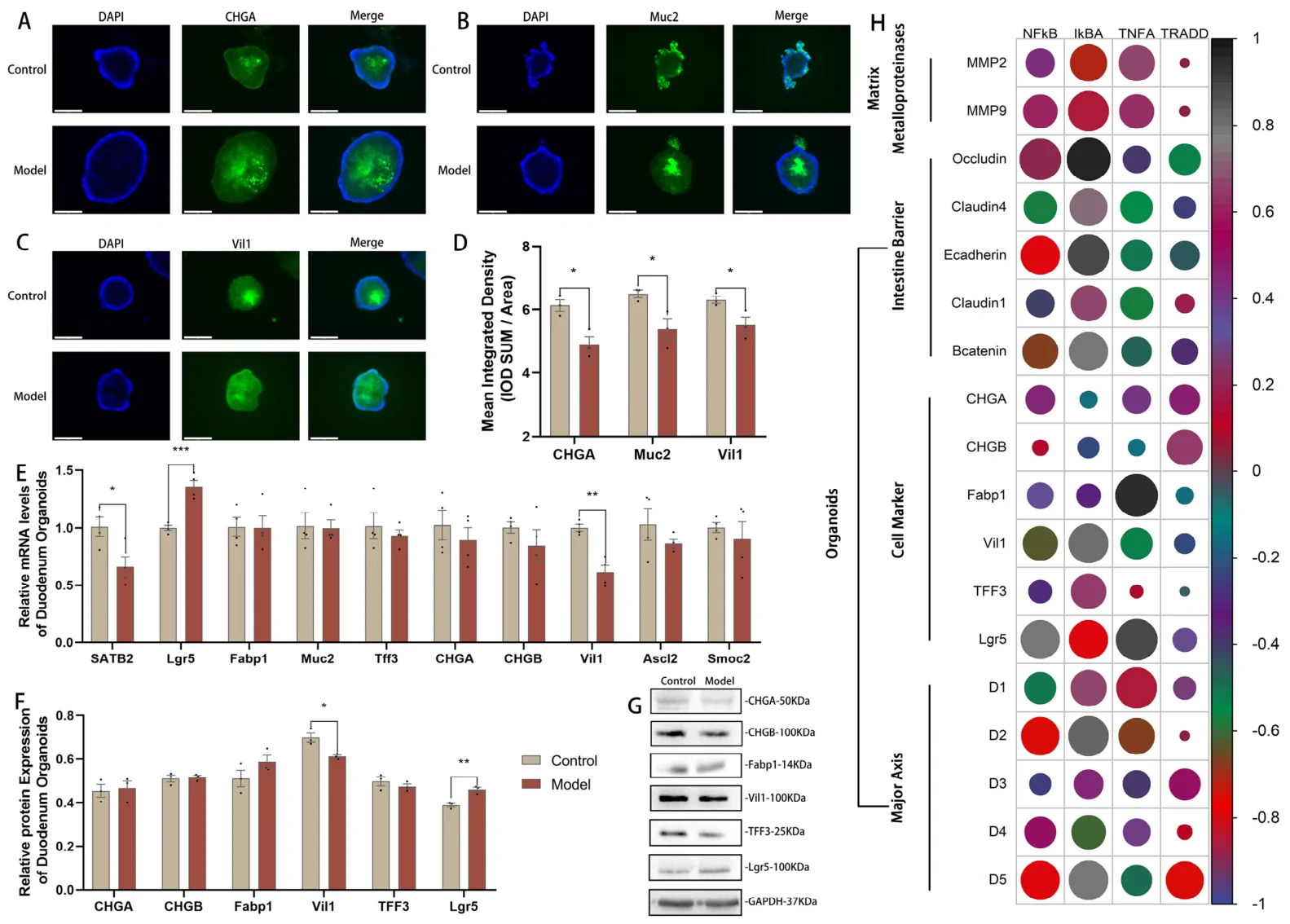
The expression of cell marker protein in intestine stem cells. (**A**–**C**), representative immunofluorescence images of the organoids labeled with DAPI (blue) and target protein antibodies (green), scale bar: 150 μm; (**D**), the quantification of protein relative expression using mean integrated density, *n* = 3; (**E**), the expression of intestine cell-marker-related genes in the mRNA level, *n* = 4; (**F**,**G**), the relative protein expressions of the duodenum organoids. For each experiment, *n* refers to the number of independent biological replicates. The results are reported as mean ± SEM, *n* = 3. * *p* < 0.05, ** *p* < 0.01, and *** *p* < 0.01. (**H**), the correlation between the NF-κB pathway and other indicators.

To further study the stemness of the intestinal stem cells after cardiac surgery, the representative immunofluorescence analysis of stem cell markers is presented in Figure 6A. Quantitative analysis using mean integrated density revealed significantly reduced expression of the enteroendocrine marker CHGA (*p* = 0.0171), the goblet cell marker Muc2 (*p* = 0.0341) and the enterocyte marker Vil1 (*p* = 0.0488) in the model group compared with the control group (Figure 6D). The mRNA analysis demonstrated a global downregulation of stem-cell-associated markers, with statistically significant reductions in SATB2 (*p* = 0.0263) and Vil1 (*p* = 0.0011). Conversely, the model group exhibited elevated Lgr5 expression (*p* = 0.0008), indicative of enhanced stem cell maintenance. Western blot validation confirmed these findings, showing decreased Vil1 (*p* = 0.0167) and increased Lgr5 (*p* = 0.0053) protein expression in the model group. Integrated analysis of the experimental data revealed significant inverse correlations between NF-κB pathway activation and intestinal barrier integrity markers in organoid models (Figure 6H). Furthermore, the physical characteristics of the organoids also showed a negative correlation with the NF-κB pathway.

### 3.7. Cardiac-Surgery-Induced Impairment of Intestinal Stem Cell Proliferative Capacity

**Figure 7:**
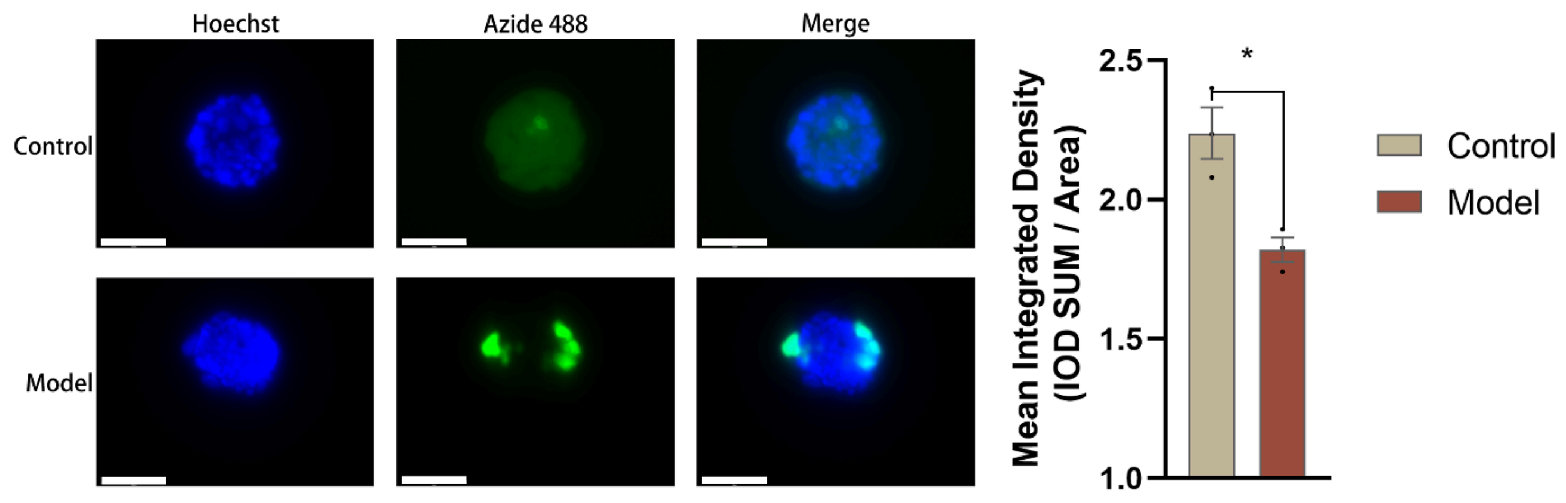
The proliferation capacity of the intestinal stem cells isolated from post-cardiac-surgery intestine segments. The proliferate ability was confirmed by 5-ethynyl-20-deoxyuridine (EdU) staining, the scale bar: 50 μm. The quantification of protein relative expression using mean integrated density. For each experiment, *n* refers to the number of independent biological replicates. The results are reported as mean ± SEM, *n* = 3. * *p* < 0.05.

The proliferative capacity within the organoid was confirmed by 5-ethynyl-20-deoxyuridine (EdU) staining (Figure 7), indicating that proliferating cells were present throughout the organoids under this culture condition. Quantitative analysis of the proliferative capacity based on mean integrated density further showed that the organoids in the control group had greater proliferative capacity than those in the model group (*p* = 0.0151).

## 4. Discussion

Cardiac surgery induces profound systemic stress during clinical procedures, with emerging evidence suggesting that prolonged surgical trauma triggers inflammatory cascades through mechanisms involving blood–brain barrier disruption and subsequent multi-organ dysfunction [22]. Gastrointestinal complications following cardiac surgery present significant diagnostic challenges and carry substantial mortality risks [23,24]. In a retrospective cohort analysis of 29,909 cardiac procedures, Elgharably [25] identified gastrointestinal complications in 1037 cases. Mechanistically, systemic vasoconstriction and blood flow redistribution during cardiopulmonary bypass preferentially divert circulation from the splanchnic circulation. This hemodynamic shift precipitates splanchnic hypoperfusion, culminating in intestinal mucosal ischemia and hypoxia. Subsequent reperfusion during cardiopulmonary bypass weaning and blood product administration induces ischemia–reperfusion injury, paradoxically aggravating mucosal damage. The gastrointestinal system demonstrates particular vulnerability to such stressors, ultimately leading to impaired intestinal barrier integrity. This pathophysiological cascade causes intestinal epithelial barrier dysfunction via inflammatory-mediated tight junction protein dysregulation. Existing research on gastrointestinal complications is predominantly retrospective, which not only limits progress in identifying high-risk patients but also delays understanding of the mechanisms underlying gastrointestinal complications after cardiac surgery. Therefore, further study is needed.

To validate surgical stress induction, we quantified systemic stress biomarkers in porcine models post-cardiopulmonary bypass. ACTH concentrations serve as a validated biomarker for physiological stress monitoring, with extensive applications in clinical research [26,27]. In response to external stress, the activation of the hypothalamic–pituitary–adrenal (HPA) axis and sympathetic nervous system stimulates ACTH secretion, triggering adaptive neuroendocrine responses to environmental challenges through glucocorticoid-mediated pathways [28]. Our results revealed significant ACTH elevation following cardiac surgery, confirming the induction of systemic surgical stress in porcine models. In addition to confirming systemic stress induction, the elevated ACTH levels observed in the present study may partially explain the concomitant increase in myocardial injury biomarkers. Cardiac surgery, particularly under ischemia–reperfusion conditions, is known to trigger neuroendocrine activation together with inflammatory and oxidative responses, all of which may contribute to myocardial injury. In this context, the parallel elevation of ACTH and myocardial injury markers in our study suggests a possible association between postoperative stress activation and myocardial damage. However, the present study was not designed to establish a direct causal relationship between stress hormone signaling and myocardial injury. This interpretation is generally consistent with previous clinical studies showing that cardiac surgery and cardiopulmonary bypass are associated with intestinal injury and barrier dysfunction [10,29].

We subsequently analyzed the mRNA expression of intestinal-barrier-associated genes, and the downregulation of these genes suggests impaired barrier function. Clinical evidence indicates cardiac procedures impose significant physiological stress on the intestinal barrier [3,30]. The intestinal barrier’s structural integrity is principally maintained by tight junctions including ZO-1 and Occludin proteins, which regulate paracellular permeability through dynamic intercellular interactions [31]. Our findings corroborate this structural vulnerability. Nevertheless, the precise molecular mechanisms underlying cardiac-surgery-induced intestinal barrier impairment remain to be fully elucidated.

A critical pathophysiological consequence of gastrointestinal barrier dysfunction involves endotoxin translocation and subsequent systemic inflammation, as demonstrated in porcine models of intestinal ischemia [32]. Clinical observations consistently associate prolonged cardiopulmonary bypass durations with systemic inflammatory response syndrome (SIRS) and multi-organ dysfunction [33,34]. Emerging therapeutic strategies employing a double-cell-targeting system to inhibit NF-κB signaling have shown efficacy in restoring intestinal barrier integrity through cytokine modulation [35]. Building on this evidence, we investigated NF-κB activation patterns during cardiac surgery. Quantitative analysis of both transcriptional and translational levels demonstrated sustained activation of the NF-κB pathway during and after the procedure, suggesting a mechanistic link between pathway activation and surgical-stress-induced intestinal compromise.

Furthermore, the concomitant upregulation of matrix metalloproteinase (MMP) family members further supported NF-κB-mediated inflammatory cascades. MMPs, known regulators of extracellular matrix remodeling in physiological processes ranging from embryogenesis to inflammation [14,36], are usually used as effective indicators of organ damage [37,38], and may play an important role in mediating surgery-associated intestinal pathophysiology. For example, MMP-9 mediates detrimental effects through pathological extracellular matrix degradation, inflammatory mediator activation and microvascular permeability [39]. However, its early-phase post-reperfusion upregulation also exerts tissue-protective functions via the coordinated removal of necrotic debris, the liberation of growth factors/cell surface receptors, provisional scar matrix remodeling, inflammatory signal modulation, and angiogenic regulation [40,41]. This time-restricted MMP-9 elevation likely represents an adaptive mechanism that promotes faster wound resolution. In our study, the upregulation of MMP-9 and MMP-2 may indicate an abnormal intestinal environment.

To investigate postoperative intestinal pathophysiology, crypt-derived intestinal stem cells were isolated for three-dimensional organoid culture. These porcine intestinal organoids served as a physiologically relevant ex vivo epithelial model for evaluating the effects of surgical stress, as intestinal organoids have been widely used to study epithelial injury, regeneration, and ischemia/reperfusion-related responses [42]. In the present study, cardiac surgery appeared to impair organoid development, possibly through a cascade of postoperative intestinal injury and stress-related responses, as reflected by delayed crypt formation in the surgical group compared with the controls. For example, porcine jejunal organoids in the control group developed more complex tube-like and budding structures earlier than those in the model group. To further characterize these phenotypic differences, we quantified major axis, minor axis, and roundness as morphometric descriptors of organoid architecture. In intestinal organoid systems, such shape-related parameters are commonly used to capture changes in organoid size, symmetry breaking, budding behavior, and structural maturation, rather than serving as stand-alone indicators of proliferation [43,44]. Thus, the greater major and minor axes observed in control organoids, together with the alterations in roundness, suggest more advanced growth and morphogenesis, whereas the surgical group displayed impaired organoid development and delayed architectural remodeling.

Further investigations were conducted here to delineate molecular alterations associated with cardiac-surgery-related intestinal injury in these organoids. As a new model in vitro, organoids enable the precise investigation of epithelial pathophysiology [45,46]. Previous studies also showed that cardiac surgery, particularly when associated with cardiopulmonary bypass, may induce intestinal mucosal injury, endotoxemia, dysbiosis, and barrier dysfunction [10,29]. Therefore, organoids established from postoperative intestinal tissues may provide an ex vivo platform for assessing epithelial-intrinsic injury and regenerative responses after surgical stress. In our study, immunofluorescence staining demonstrated the altered localization and expression of proteins related to the epithelial barrier, and quantitative analyses further showed the downregulation of tight junction proteins and related genes in the model group. These findings indicate that cardiac-surgery-induced intestinal stress compromises epithelial barrier integrity and function.

Next, expressions of epithelial cell markers at both the mRNA and protein levels were evaluated. Our results revealed enhanced multilineage differentiation capacity in control organoids, as evidenced by a broader spectrum of mature epithelial cell populations compared with surgical models. In contrast, organoids from the surgical group exhibited concomitant upregulation of Lgr5 at both the transcriptional and translational levels, suggesting preservation of the stem cell compartment but impaired differentiation dynamics. Quantitative morphometric analysis also indicated reduced developmental capacity in the model group relative to the controls. Taken together, these findings suggest that surgical stress compromises intestinal epithelial homeostasis and attenuates organoid development. Notably, the delayed but progressive morphogenesis observed during extended culture suggests that the stem cell impairment may be transient rather than irreversible, indicating a degree of phenotypic plasticity.

Nevertheless, several limitations should be acknowledged. First, intestinal organoids primarily recapitulate the epithelial compartment and do not fully reproduce the vascular, immune, neural, and microbial components of the in vivo intestinal niche [47,48]. Second, major/minor axis and roundness provide indirect morphological information on organoid growth and remodeling, but they are not equivalent to direct proliferation assays. Third, although our findings support an association between cardiac-surgery-related intestinal injury and altered epithelial regenerative behavior, the present evidence remains largely correlative, and additional intervention experiments are needed to further verify the causal role of the proposed NF-κB/MMP axis. Fourth, although changes in barrier-related markers were observed, a direct functional assessment of intestinal permeability was not performed in the present study. Therefore, further studies incorporating mechanistic inhibition experiments and functional permeability assays are warranted to strengthen and extend the current findings. From a translational perspective, these findings suggest that cardiac-surgery-related intestinal injury may involve both epithelial barrier disruption and impaired regenerative responses, thereby highlighting the intestine as a potential target for perioperative monitoring and intervention. Nevertheless, the direct extrapolation of these findings to clinical practice should be made with caution and requires further validation in human studies.

## 5. Conclusions

**Graph 1:**
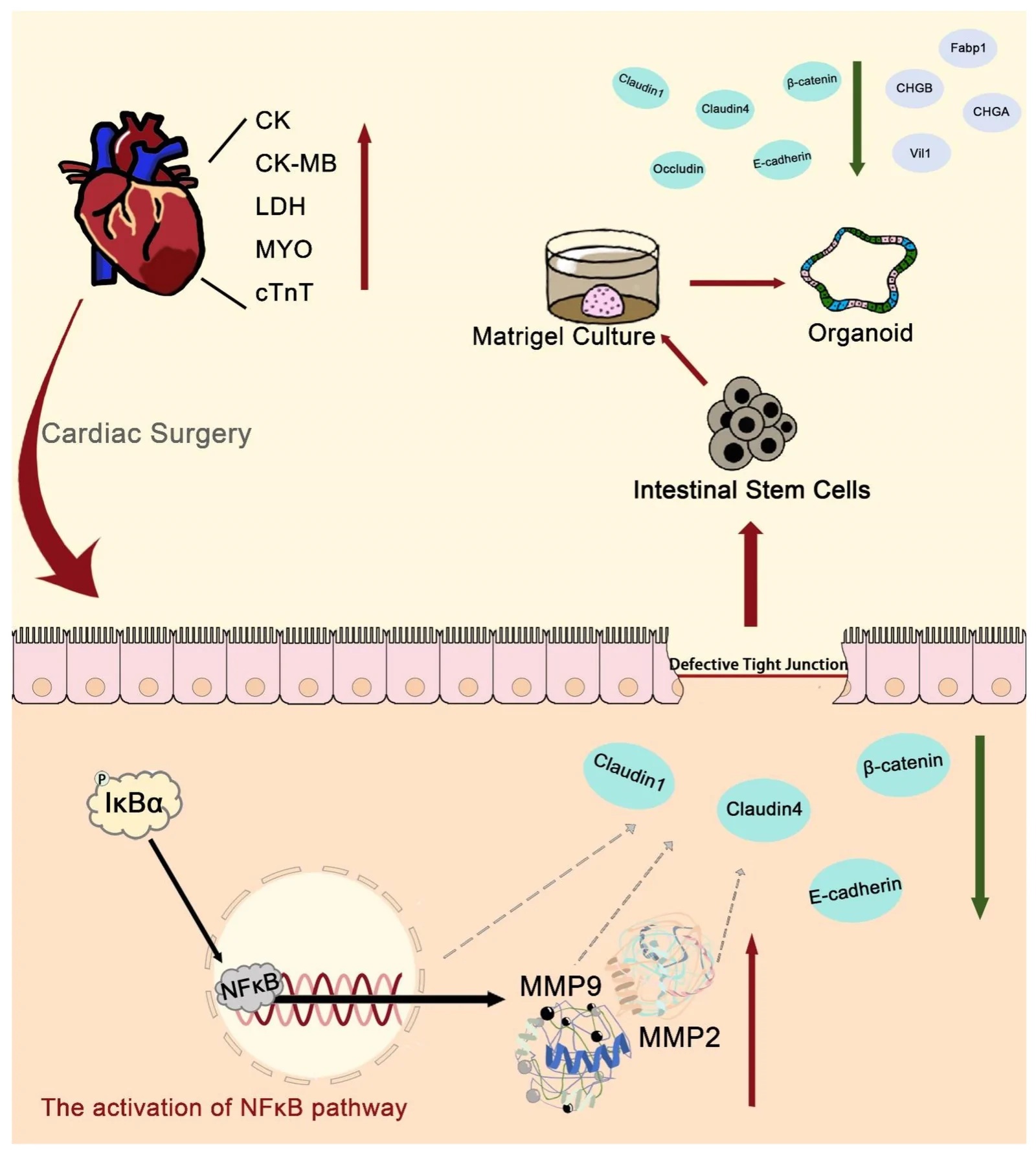
Introduction.

Collectively, our findings elucidate the postoperative intestinal pathophysiology in animals undergoing cardiac surgery, delineating the molecular mechanisms and potential signaling pathways involved. We systematically investigated the impact of cardiac surgery on animal intestinal health, quantifying the expression profiles of epithelial barrier components and specific cell markers within jejunal organoid models derived from these animals. Quantitative analyses revealed impaired proliferative dynamics and delayed crypt–villus morphogenesis in organoids cultured from the surgical group during the early phase; however, these organoids demonstrated preserved differentiation capacity after extended culture (Graph 1). These mechanistic insights establish a foundational framework for translational investigations into surgical-stress-associated gastrointestinal complications in animals and directly inform clinical veterinary research aimed at improving postoperative care and outcomes.

## Data Availability

The original contributions presented in this study are included in the article/Supplementary Material. Further inquiries can be directed to the corresponding author(s).

## Supplementary Materials

The following supporting information can be downloaded at: https://www.mdpi.com/article/10.3390/biology15120930/s1. Table S1. Primers used in this study. Table S2. Antibodies used in this study.

## Abbreviations

SEM	standard error of the mean
CK	creatine kinase
CK-MB	creatine kinase-MB
MYO	Myoglobin
cTnT	Elecsys Troponin T hs STAT
LDH	lactate dehydrogenase
DAPI	4′,6-diamidino-2-phenylindole
EDTA	ethylene diamine tetraacetic acid
PBS	phosphate-buffered saline
ZO-1	zonula occludens-1
Claudin1	CLDN-1
Claudin4	CLDN-2
SATB2	SATB homeobox 2
Vil1	villin 1
Fabp1	fatty acid binding protein 1
Muc2	mucin 2
Tff3	trefoil factor 3
CHGA	chromogranin A
CHGB	chromogranin B
Lgr5	leucine rich repeat containing G protein-coupled receptor 5
Ascl2	achaete-scute family bHLH transcription factor 2
Smoc2	SPARC related modular calcium binding 2
