# Supplementary material for: Intestinal Barrier Dysfunction and Stem Cell Impairment Following Cardiac Surgery in Pigs: A Porcine Model Study
Source: Biology (Basel). 2026 Jun 15;15(12):930. doi: 10.3390/biology15120930 (PMC13295967; doi:10.3390/biology15120930)
Supplement: Supplementary file 1 [file biology-15-00930-s001.zip › biology-4175420-supplementary.pdf]

Table S1. Primers used in this study.

**Gene**	**Forward Primer**	**Reverse Primer**	NCBI ID
Occludin	CAGGTGCACCCTCCAGATTG	ATGTCGTTGCTGGGTGCATA	NM_001163647.2
ZO-1	TCAAGGTCTGCCGAGACAAC	ATCACAGTGTGGTAAGCGCA	XM_021098856.1
Claudin1	ATGACCCCAGTCAATGCCAG	CCCTCTCCCCACATTCGAGA	NM_001244539.1
Claudin4	ATGCAGTGCAAGGTGTACGA	GAGTAGGGCTTGTCGGTACG	NM_001161637.1
E-Cadherin	CGACGGTGTGGTTACAGTCA	GTCACCGTGAGTGTGGTGAT	NM_001163060.1
β-catenin	GAGACGGAGGAAGGTCCGAG	ATTGCACGTGTGGCAAGTTC	NM_214367.1
SATB2	TGTGGGAGTGAACACCGTC	CAATAAAACGCGCAGGGACC	XM_021076069.1
Vil1	CAGCTTGCCACAACTCCTGA	AGCAGTCACCGTCGAAGAAG	XM_001925167.6
Fabp1	AGGGGACATCGGAAATCGTG	TCACACTCCTCTCCCAAGGT	NM_001004046.2
Muc2	CGGTCAAGGACGACACCATC	GTTCCCAGGGCAGCTCTTAG	XM_021082584.1
Tff3	CTGAAGTGACCACGCTTCCT	GCTTCTCAAGGGTCACGGAA	NM_001243483.1
CHGA	ACTCCGAGGAGATGAACGGA	GCGAGGTCTTGGAGCTCTTT	NM_001164005.2
CHGB	CGCGAGGAATCTGAGGAGTC	TGCTCCCCGGAGTCATAGAA	NM_214081.2
Lgr5	AGCCTTTGTAGGCAACCCTT	GAAGGTGTACACTGCACGGA	NM_001315762.1
Ascl2	CTGACCAAGGGCTAGTGTGG	CTCGTCAAGCCTCCAAGTGT	NM_001122991.1
Smoc2	ACAGTCTCCTTGCAAATCTTTTCC	CGTCCAACACACTCGTCAGA	XM_021085691.1
β-actin	AGAAGCTGTGATGGACGCAG	ACCCCTGGGAGTTGTACCTT	XM_021086047.1
NF-κB	CCAGACCAACAACAACCCCTTCC	AAGCAGAGCCGCACAGCATTC	NM_001114281.1
IκBα	TGGTGTCGCTCTTGTTGAAGTGTG	GCTGCTGTATCCGAGTGCTTGG	NM_001005150.1
TNF-α	GCACTGAGAGCATGATCCGAGAC	CGACCAGGAGGAAGGAGAAGAGG	NM_214022.1
TRADD	ACCGCAGGGTTAATAGGGC	CCATTCTGCCTCCTTGTCCA	XM_021094050.1

Table S2. Antibodies used in this study.

Primary antibodies	Dilution ratio
Claudin 1	1:300
Claudin 4	1:300
Occludin	1:300
β-Catenin	1:300
E-cadherin	1:300
Vil1	1:300
CHGA	1:300
CHGB	1:250
Fabp1	1:300
TFF3	1:400
Lgr5	1:150
NF-κB	1:100
p-NF-κB	1:100
IκBα	1:100
p-IκBα	1:100
MMP2	1:100
MMP9	1:100
GAPDH	1:500
